# Supplementary material for: A Global Screen for Assembly State Changes of the Mitotic Proteome by SEC-SWATH-MS
Source: Cell Syst. 2020 Feb 26;10(2):133–155.e6. doi: 10.1016/j.cels.2020.01.001 (PMC7042714; doi:10.1016/j.cels.2020.01.001)

Q9H3K2 | GHITM_HUMAN | GHITM DERP2 MICS1 TMBIM5 My021 UNQ244/PRO281
Monomer MW [kDa]: 37.205 Monomer expected elution fraction: 49

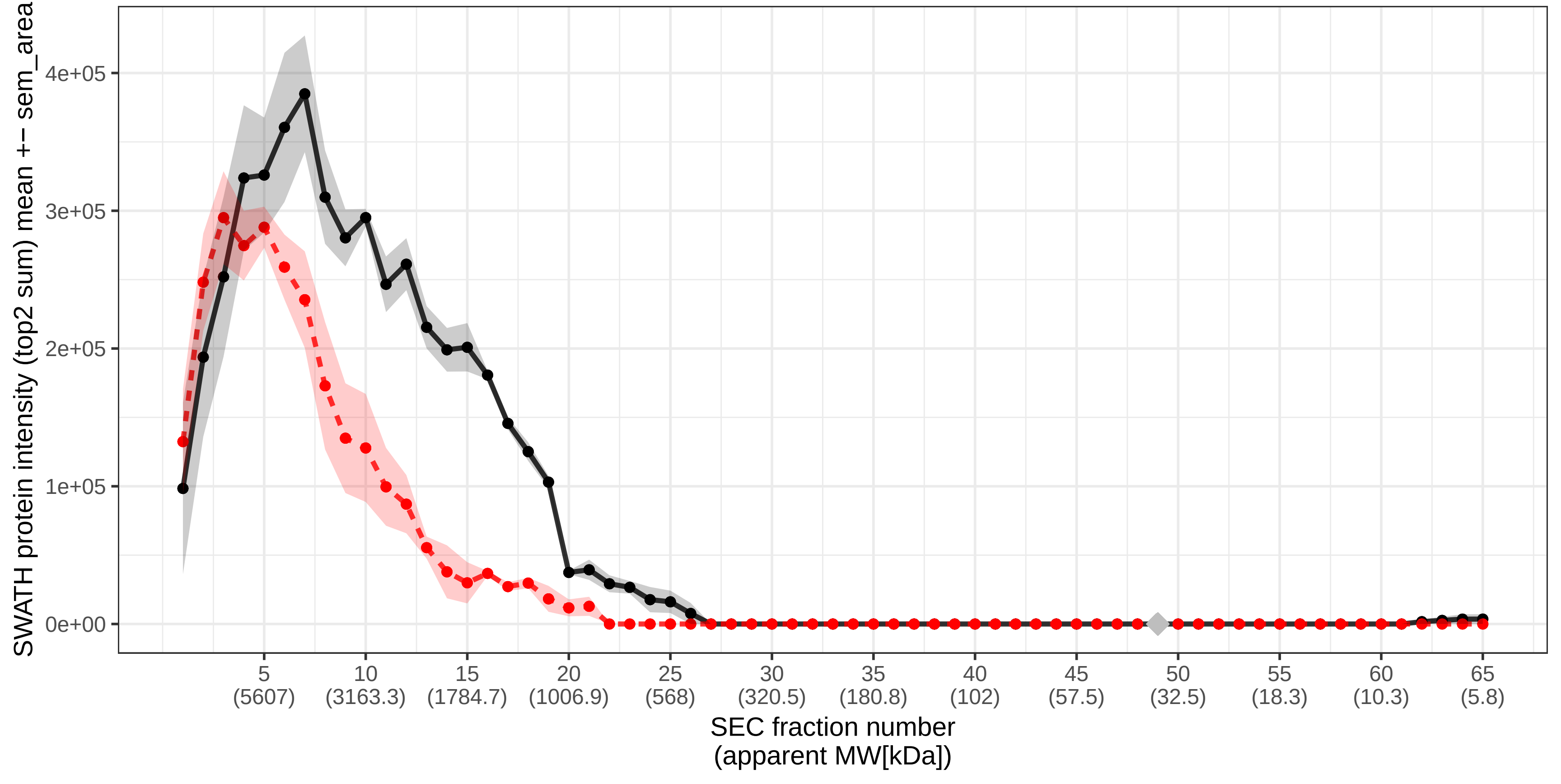

Supplement: Data S1. SEC-SWATH-MS Protein Chromatograms, Related to Figure 1 [file mmc6.zip › SECchrom_Q9H3K2_GHITM_HUMAN_GHITM_DERP2_MICS1_TMBIM5_My021_.pdf]
